# A Functional Trait‐Based Approach to Mapping Climate‐Driven Changes in Temperature‐Dependent Feeding Suitability

**DOI:** 10.1002/ece3.73623

**Published:** 2026-05-03

**Authors:** Guillaume Marchessaux, Sadi Aksu, Ali Serhan Tarkan

**Affiliations:** ^1^ Aix Marseille Univ, Université de Toulon CNRS, IRD, MIO Marseille France; ^2^ Vocational School of Health Services Eskişehir Osmangazi University Eskişehir Türkiye; ^3^ Department of Ecology and Vertebrate Zoology, Faculty of Biology and Environmental Protection University of Lodz Lodz Poland; ^4^ Department of Basic Sciences, Faculty of Fisheries Muğla Sıtkı Koçman University Muğla Türkiye

**Keywords:** climate change, ecological risk assessment, functional traits, species distribution modeling, thermal performance curves, trophic interactions

## Abstract

Climate change is altering species distributions and trophic interactions, necessitating predictive tools for assessing future ecological impacts. This study applies thermal performance curves (TPCs) to project temperature‐dependent changes in relative feeding suitability across a set of freshwater, marine, and terrestrial species under baseline and mid‐century climate conditions. Using a normalized Thermal Habitat Suitability (THS) index, we mapped spatial patterns of potential feeding performance and categorized them into five standardized suitability classes (0–1 range). Results indicate that, within the limited sample analyzed, freshwater species tended to show increased feeding efficiency, while marine and terrestrial species exhibited more variable trends. The study emphasizes the necessity of integrating prey distribution modeling to account for spatial match‐mismatch dynamics under climate change. Overall, this approach provides a temperature‐based decision‐support tool that can help managers prioritize species for monitoring based on projected shifts in feeding suitability under future climate conditions.

## Graphical abstract

This study integrates thermal performance curves with species distribution modeling to assess how climate‐driven temperature changes affect feeding efficiency across freshwater, marine, and terrestrial species. By mapping thermal habitat suitability and projected feeding‐rate shifts, we develop a priority ranking framework to identify species and regions most vulnerable to climate change. The results provide a scalable decision‐support tool for guiding conservation and ecosystem management under future climate scenarios.

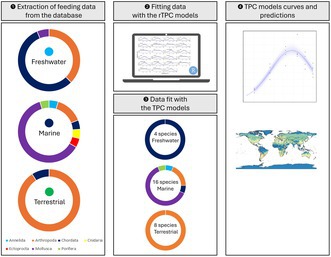

## 1 Introduction

In 2011, Englund et al. (2011) demonstrated the importance of temperature in shaping functional responses, defined as the relationship between a predator's consumption rate and prey density (Petersen and DeAngelis 1992). Functional responses have long been recognized as a cornerstone of predator–prey theory, with early work highlighting their role in regulating population and community dynamics (Nicholson 1935; Holling 1959). Subsequent research has explored a wide range environmental drivers influencing functional responses, resulting in the development of numerous model formulations (Jeschke et al. 2002). More recently, a growing body of work has examined how temperature affects predator–prey interactions, including effects on interaction strength, metabolic rates, and food web dynamics (e.g., Rall et al. 2010; Dell et al. 2014; Gomides et al. 2021; Carroll et al. 2024). However, despite these advances, the integration of temperature‐dependent interaction processes into spatially explicit predictive frameworks remains limited. In particular, most large‐scale ecological forecasting approaches, such as species distribution models (SDMs), still rely primarily on correlative relationships and rarely incorporate mechanistic representations of how temperature shapes trophic interactions. This creates a key gap in our ability to predict how climate‐driven changes in temperature will translate into changes in interaction strength and, ultimately, ecological impact across spatial scales.

Interactions between consumers and their resources shape the structure of food webs and are a fundamental component of ecological systems. While these interactions can be quantified in numerous ways (Wootton and Emmerson 2005; Novak and Wootton 2010), functional responses are widely used to formalize feeding interactions, describing how consumer foraging rates change with resource density (Holling 1959). Beyond characterizing species interactions, functional responses are commonly used to interpret food web links (McCann 2011), understand population dynamics (Yodzis and Innes 1992), and inform biocontrol strategies (Uiterwaal and DeLong 2018). Quantitative variation in functional responses influences the stability, dynamics, and abundances of consumers and resources across ecosystems (Yodzis and Innes 1992; Weitz and Levin 2006; Petchey et al. 2008; Gilljam et al. 2011; McCann 2011; DeLong and Vasseur 2012; Pawar et al. 2012; Brose et al. 2019). However, translating full functional response formulations into spatially explicit predictions remains challenging, as parameters such as attack rate and handling time are rarely available across broad environmental gradients (Dell et al. 2014; Brose et al. 2019). In this context, feeding efficiency that is expressed as realized consumption rates across prey densities and temperatures provides a tractable proxy for interaction strength (Petchey et al. 2008; DeLong and Vasseur 2012). This can be more readily integrated with spatial predictors, while retaining key aspects of predator–prey dynamics.

Climate change is fundamentally reshaping species distributions. As temperatures rise, suitable habitats shift, causing species to expand into new regions while disappearing from others (Goicolea et al. 2025; Fialas et al. 2025). This global redistribution of life presents significant challenges for biodiversity, ecosystem stability, food security, and human well‐being (Pecl et al. 2017). In response, numerous studies have sought to understand the mechanisms driving species movements, measure rates and magnitudes of range shifts (Chen et al. 2011; VanDerWal et al. 2013), and predict future distributions under changing climate scenarios (Emiroğlu et al. 2023; Di Febbraro et al. 2023; Restrepo‐González et al. 2026). However, currently many SDMs rely on basic correlation between species and environmental conditions like temperature (Wisz et al. 2013). These often lack an explicit mechanistic representation of how thermal sensitivity constrains species interactions. Hybrid approaches may partially address this limitation by incorporating physiological thresholds or dispersal processes, yet they typically do not capture how temperature‐dependent changes in interaction strength between predators, prey and competitors influence redistribution dynamics (Buckley et al. 2011). Conversely, dynamic food‐web models can represent temperature‐dependent trophic interactions under climate change scenarios, however, they generally require extensive parameterisation and are rarely transferable across large spatial scales relevant for biogeographic projections.

Thermal performance curves (TPCs) provide a complementary framework for linking temperature‐dependent physiological performance to interaction strength and habitat suitability within spatially explicit distribution models. Integrating TPC‐derived constraints into SDMs therefore offers a tractable way to incorporate biologically meaningful temperature sensitivities into range‐shift projections while retaining the spatial scalability required for predicting redistribution under climate change (Bosso et al. 2026). Such integration can improve predictions of community reassembly by capturing how climate change alters not only where species can persist but also how their interactions are likely to change across shifting environmental gradients (Tylianakis et al. 2008; Schmitz et al. 2017).

Climate plays a key role in shaping species distributions (Hampe 2004), making it critical to incorporate both present and future climate conditions when modeling species range shifts (Heikkinen et al. 2006). This challenge is further complicated in regions where non‐native species are introduced and spread due to warming (Searcy et al. 2023; Tarkan et al. 2024). When these species establish in local communities, they may intensify ecological pressures on native species (Radinger et al. 2019; Radinger and García‐Berthou 2020). To manage these shifting dynamics, researchers and practitioners need tools that accurately capture the spatial and temporal nature of species responses to environmental change.

Species Distribution Models (SDMs) are widely used to predict future species distributions and provide early‐warning systems for biodiversity threats (Elith and Leathwick 2009; Rathore and Sharma 2023). These models typically correlate species' observed distributions with environmental variables to infer habitat suitability and predict future range shifts (Sarà et al. 2018; Aksu et al. 2024). While traditional correlative SDMs rely primarily on statistical associations between species presence and environmental predictors, more recent modeling frameworks increasingly incorporate physiological proxies, dispersal constraints and demographic processes to improve ecological realism. Nevertheless, many SDMs still represent species responses to temperature indirectly through environmental correlations rather than explicitly modeling how temperature‐dependent changes in biological performance influence trophic interactions and species dynamics at local scales (Gilbert et al. 2014; Martínez et al. 2015). Mechanistic niche models, such as biophysical models (Kearney and Porter 2009; Buckley et al. 2011), partially address this limitation by relating environmental variables to the energy balance and performance of the organisms. These models usually apply to single species and are less frequently used to represent temperature‐dependent variation in the strength of interactions between predators and prey along spatial gradients. A different and complementary approach is to use functional trait‐based mechanistic models that integrate species‐specific physiological responses such as temperature‐dependent feeding efficiency into spatial prediction frameworks (Cooke 2014; Bosch‐Belmar et al. 2021). By explicitly incorporating temperature effects on interaction performance rather than relying solely on inferred habitat suitability, this approach provides a quantitative pathway to link climate‐driven environmental change with trophic dynamics and species redistribution. However, while correlative SDMs may lack precision in fine‐scale ecological predictions, mechanistic models are often computationally demanding and data‐intensive, making them challenging for large‐scale applications (Buckley et al. 2011; Marn et al. 2020). A promising direction for improving predictive performance involves hybrid modeling approaches, which integrates mechanistic information into correlative SDMs (Kearney and Porter 2009; Bosch‐Belmar et al. 2021). Bayesian techniques provide a robust framework for incorporating species' physiological data into stochastic SDMs (Gamliel et al. 2020), although such approaches are not implemented here. Instead, our study incorporates temperature‐dependent feeding efficiency derived from thermal performance relationships as an additional mechanistic constraint on species interactions. This approach provides a tractable way to account for temperature‐driven variation in predator–prey interaction strength, an essential but often overlooked component of climate change impacts on ecological dynamics (Elith and Leathwick 2009). This combined approach improves our ability to model species' responses to temperature shifts, particularly in relation to feeding efficiency—an essential but often overlooked aspect of climate change impacts on predator–prey dynamics.

**FIGURE 1:**
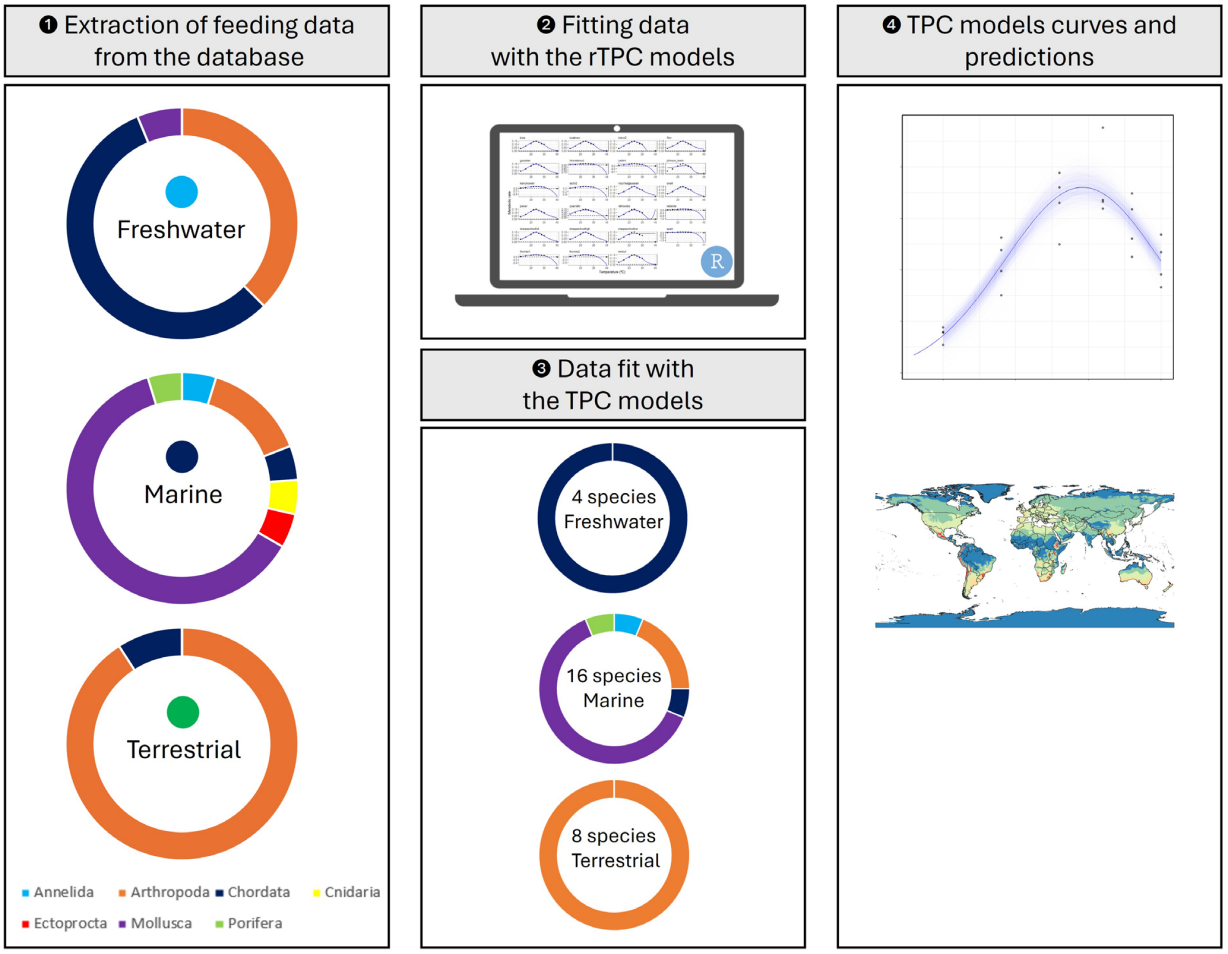
Workflow of the study and species sorting for modeling.

Here, we developed and applied an integrated modeling framework that combines correlative SDMs with mechanistic temperature‐dependent functional trait information to assess how climate‐driven changes in thermal conditions influence predator feeding efficiency across freshwater, marine, and terrestrial environments (Figure 1). Specifically, we test whether incorporating temperature‐dependent interaction performance improves spatial predictions of consumption‐rate patterns compared with suitability estimates derived from SDMs alone. We then project current and future distributions of the focal species and classify predicted consumption rates into five functional levels to assess how trophic interaction intensity is expected to shift under future climate scenarios. Ultimately, our aim is to demonstrate how integrating temperature‐dependent interaction performance into spatial prediction frameworks can support ecologically informed management and spatial planning under climate change.

## 2 Materials and Methods

### 2.1 Sources of Thermal Performance Datasets

To assess the effect of temperature quantitatively with regard to feeding processes, we retrieved data from the Figshare resource “No universal mathematical model for thermal performance curves across traits and taxonomic groups” (Kontopoulos et al. 2024). From this dataset, we included only data from animal species and traits directly associated with feeding, such as ingestion rate, foraging rate, prey capture efficiency, and digestive rate. Traits unrelated to feeding—such as growth rate, reproduction, or general metabolism—were excluded. When classifying traits, we relied on the definitions provided by the original dataset and supplemented them with functional interpretations from primary literature to ensure that only feeding‐relevant traits were retained. Additionally, we assessed data quality across taxa by excluding entries with missing temperature ranges, unrealistic thermal performance values (e.g., negative ingestion rates), or inconsistencies in replicates, ensuring that our analysis reflected reliable, comparable measurements across species.

### 2.2 Feeding Efficiency Predictions

The Thermal Performance Curves (TPCs) were modeled with R (v. 4.5.2.), utilizing the package rTPC (Padfield, O'Sullivan, and Windram 2026). For every species, 22 possible nonlinear models available in the package were scored to select the best model with the lowest value of Akaike's Information Criterion (AIC), following common criteria (Padfield, O'Sullivan, and Pawar 2021; Bosch‐Belmar et al. 2022; Marchessaux et al. 2022). To reduce the risk of overfitting, we evaluated model convergence and parameter identifiability by excluding species if fitting failed or produced unrealistic parameter estimates. Parameter uncertainty was quantified from the model fits and propagated through subsequent predictions of feeding efficiency, ensuring that downstream analyses reflect uncertainty in TPC estimates. These procedures provide both model reliability and transparency regarding limitations associated with small sample sizes and complex nonlinear forms. Species were excluded if no model converged, often due to insufficient observations or inadequate temperature coverage, resulting in poor parameter estimation.

Those species for which model parameter estimation proved poor, often due to a lack of observations or inadequate temperature extent, were removed from analysis. Thus, 28 species were selected for spatial projection (freshwater species = 4, marine species = 16, and terrestrial species = 8) to demonstrate a model procedure. Although *Salmo salar* is an anadromous species, for the purposes of this study we classified it as a freshwater species, reflecting the primary environment where feeding data were collected and most relevant to thermal performance analyses. We emphasize that this selection serves as a proof‐of‐concept, constrained by data availability, and does not represent a comprehensive global assessment.

The generation of TPCs for well‐fitted species was followed by visualization estimation of the uncertainty using bootstrapped 95% confidence intervals around fitted curves. From each TPC, key parameters were extracted, including maximum feeding rate (r_max_), optimum temperature (Topt), sensitivity to temperature (Q10), and critical thermal limits (CT_min_ and CT_max_), using functions implemented in the rTPC package. These parameters were used to characterize species‐specific thermal responses and to constrain spatial projections (e.g., via CT_min_ and CT_max_), whereas projections themselves were based on the best‐supported fitted curves. Bootstrapped confidence intervals therefore quantify parameter uncertainty but were not explicitly propagated through spatial projections.

### 2.3 Climate‐Related Spatial Projections

Unlike correlative SDMs that infer suitability from species occurrence records and environmental predictors, our approach projects temperature‐dependent feeding rates derived from Thermal Performance Curves as a mechanistic proxy for interaction strength across spatial temperature fields. In this sense, the framework represents a trait‐based mechanistic envelope model rather than a correlative SDM, allowing spatial projections of potential ecological impact based on physiological constraints rather than inferred species distributions alone. These mechanistic relationships were then projected spatially for baseline climatological conditions (1970–2000) and mid‐century projections (2041–2060), following the SSP5‐8.5 (CMIP6) scenario as a high‐emissions reference pathway to illustrate the sensitivity of feeding efficiency to projected warming. We note that current best practices increasingly recommend the use of multiple Shared Socioeconomic Pathway (SSP) scenarios; however, here the SSP5‐8.5 (CMIP6) projection was used to provide a consistent proof‐of‐concept demonstration of the modeling framework rather than a comprehensive scenario comparison. For freshwater and terrestrial species, temperature layers were obtained from the WorldClim v2.1 CMIP6 dataset at ~10‐arc‐minute spatial resolution, representing baseline conditions (1970–2000) and mid‐century projections (2041–2060) under the SSP5‐8.5 scenario. Marine temperature projections were obtained from CMIP6 datasets accessed via the Copernicus Climate Data Store using the same scenario framework.

Thermal Habitat Suitability was calculated by scaling the predicted feeding rate at a given temperature (FR_t_) relative to the species‐specific maximum feeding rate at the thermal optimum (FR_Opt_) according to the formula:

$$\mathrm{THS}={\mathrm{FR}}_{t}/{\mathrm{FR}}_{\mathrm{Opt}}$$

This transformation produces values ranging from 0 to 1, indicating relative feeding suitability (Marchessaux et al. 2022, 2024), and allows comparison of temperature‐dependent feeding performance across species with different absolute feeding rates. We note that this normalized metric does not explicitly incorporate absolute metabolic demand and therefore represents a proxy for relative feeding performance rather than absolute energetic balance. THS values were determined on a monthly basis for each cell in the world's grid and aggregated annually, providing a generalized representation of global spatial patterns in feeding suitability. Monthly temperature layers were processed at their native spatial resolution (10 arc‐minutes) without additional interpolation. Thermal Habitat Suitability (THS) values were calculated separately for each month and subsequently averaged across the 12 months to obtain annual suitability estimates for each grid cell. Climate layers representing marine and terrestrial environments were aligned to a common grid prior to analysis where necessary, and land–sea masking was applied to restrict projections to ecologically relevant environments for each species group. All raster processing steps were conducted in R using the terra package (Hijmans et al. 2026). While seasonal dynamics can strongly influence predator–prey interactions, annual aggregation was used here to illustrate the modeling framework within a proof‐of‐concept context. Spatial results were produced by applying species‐specific Thermal Performance Curve functions directly to gridded temperature layers in R, and finalized with QGIS (version 3.10.7), without additional interpolation beyond the native resolution of the climate datasets. A summary of variables and terminology used throughout the manuscript is provided in Table 1.

**TABLE 1:** Definitions of variables used in thermal performance projections.

Term	Symbol	Definition	Units	Source
Feeding rate	FR_t	Predicted feeding rate at temperature *t* derived from fitted TPC	Species‐specific (original study units)	Figshare dataset (Kontopoulos et al. 2024)
Maximum feeding rate	FR_opt	Maximum feeding rate at thermal optimum	Same as FR_t	Derived from fitted TPC
Thermal habitat suitability	THS	Normalized feeding suitability index calculated as FR_t/FR_opt	Dimensionless (0–1)	Calculated in this study
Thermal optimum	T_opt	Temperature at which FR_opt occurs	°C	Derived from fitted TPC
Lower thermal limit	CT_min	Lower temperature threshold for feeding performance	°C	rTPC output
Upper thermal limit	CT_max	Upper temperature threshold for feeding performance	°C	rTPC output
Temperature sensitivity	Q10	Rate change over 10°C interval	Dimensionless	rTPC output

### 2.4 Feeding Efficiency Change Rate

For assessing changes in suitability for feeding over time, present and projected future THS rasters were categorized into five classes: Minimal (0–0.2), Minor (0.2–0.4), Moderate (0.4–0.6), Major (0.6–0.8), and Massive (0.8–1.0). This equal‐interval classification provides a transparent and reproducible framework for summarizing spatial changes in relative feeding suitability and facilitates comparisons across species and regions. Similar standardized interval‐based classifications are commonly used in ecological suitability and risk‐mapping contexts (e.g., Landis and Koch 1977; Aksu et al. 2024). While ecological responses to temperature are continuous and may not align with discrete thresholds, this categorization was applied only for visualization and comparative spatial summaries; all suitability calculations were performed using continuous THS values. The extent of each class was measured and expressed in terms of absolute and percentage changes (Aksu et al. 2024). Rasters for present and future scenarios were separately analyzed, and mean annual suitability indices were derived for each pixel. Class assignments were based on suitability values derived from best‐supported Thermal Performance Curve fits and single‐scenario climate projections; uncertainty associated with TPC parameter estimation and climate projections was quantified separately but not explicitly propagated into categorical class assignments. Changes in classes and geographical shifts were analyzed using the terra R package and ArcGIS Pro software (version 3.4). Species were classified as “range‐expanding” when the spatial extent of areas with moderate‐to‐high Thermal Habitat Suitability (THS ≥ 0.6) increased between baseline and projected future conditions, and as “non‐expanding” otherwise. Because suitability classes represent discretised summaries of continuous THS values, spatial interpretations were supported by underlying continuous suitability metrics to avoid overemphasizing categorical shifts in management interpretation. Priority zones were defined based on the magnitude and direction of changes in annual Thermal Habitat Suitability (ΔTHS) between baseline and future projections.

## 3 Results

All the Thermal Performance Curves (TPC) are presented in Figure S1 for freshwater species, Figure S2 for marine species, and Figure S3 for terrestrial species, with model parameters summarized in Table S1.

**FIGURE 2:**
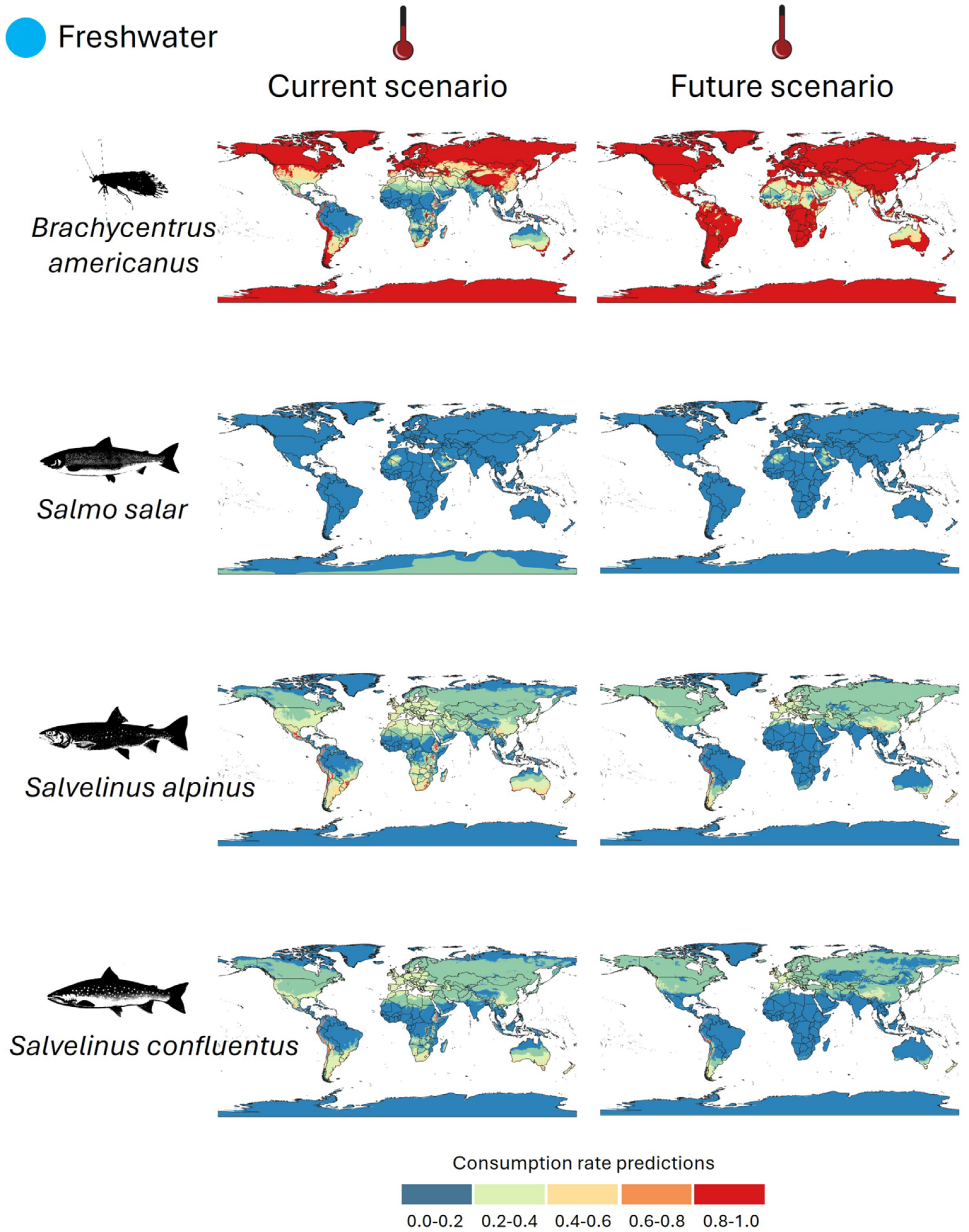
Spatial projections of relative feeding suitability (Thermal Habitat Suitability, THS) under baseline conditions (left) and mid‐century projections (right) for the freshwater species analyzed. THS values range from 0 to 1 and represent temperature‐dependent feeding suitability normalized to each species' thermal optimum.

For freshwater species, 3 fish species and 1 aquatic insect were analyzed (Figure 2). For *Salmo salar* , the current scenario shows predominantly low to moderate consumption, with higher consumption areas in North America and Europe. In the future scenario, projected feeding suitability increases across several regions, particularly in North America and Europe. *Salvelinus alpinus* currently exhibits moderate to high‐consumption in certain regions of North America, Europe, and Asia, with projected increases in feeding suitability in North America and Europe under future conditions. *Salvelinus confluentus* also shows moderate to high current consumption levels, with projections indicating a spatial expansion of higher suitability areas in North America and Europe. *Brachycentrus americanus* currently exhibits areas of moderate to high feeding suitability primarily across North America, with future projections indicating a shift toward increased suitability in higher THS classes, suggesting potential expansion of temperature‐dependent feeding performance under warming conditions. Consistent with this pattern, projections for *B. americanus* also indicate an increase in higher‐suitability areas under future conditions.

**FIGURE 3:**
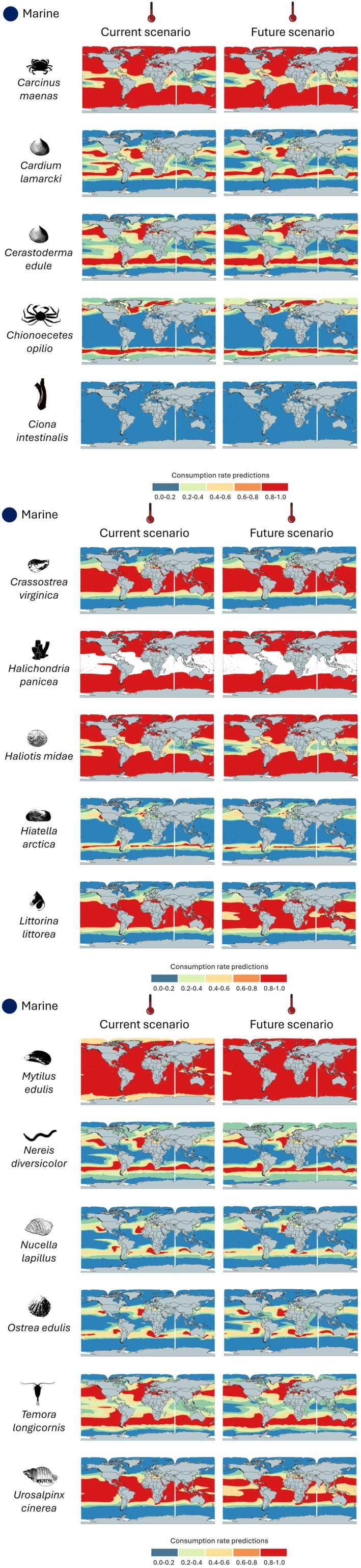
Spatial projections of relative feeding suitability (Thermal Habitat Suitability, THS) under baseline conditions (left) and mid‐century projections (right) for the marine species analyzed. THS values range from 0 to 1 and represent temperature‐dependent feeding suitability normalized to each species' thermal optimum.

For marine species, distributional patterns varied considerably among species due to their broad and differing feeding efficiency changes (Figure 3). *Mytilus edulis* exhibits a projected reduction in feeding efficiency, contracting from a widespread presence to a more restricted area, suggesting potential habitat loss. Similarly, *Nucella lapillus* and *Ostrea edulis* display comparable trends, showing a projected decline in their feeding efficiency, which may indicate increased vulnerability to environmental changes. In contrast, species such as *Carcinus maenas* and *Crassostrea virginica* demonstrate projected spatial expansion of higher suitability areas, suggesting either better adaptability or the availability of new suitable habitats under future climate conditions. Other species, including *Chionoecetes opilio* and *Halichondria panicea* , show moderate spatial shifts in projected feeding suitability, reflecting diverse ecological responses to environmental modifications.

**FIGURE 4:**
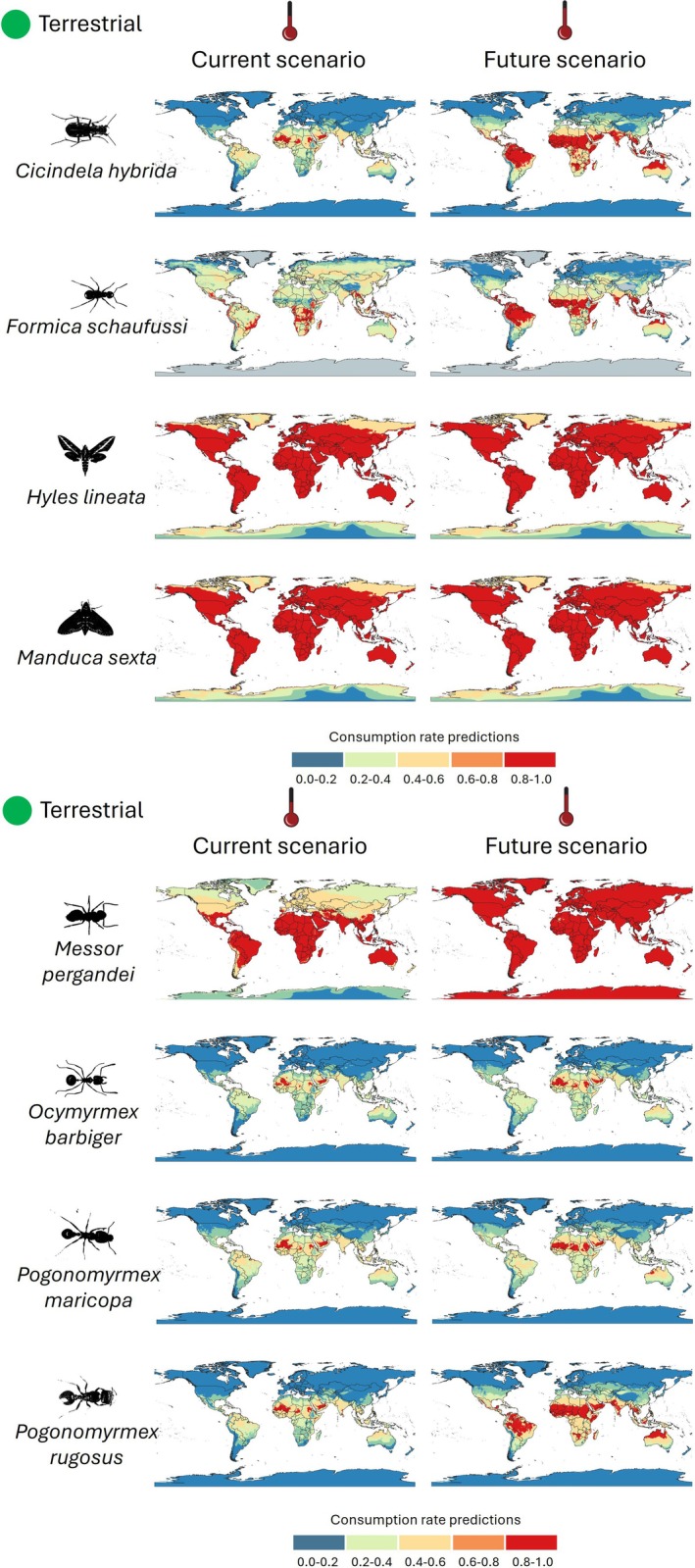
Spatial projections of relative feeding suitability (Thermal Habitat Suitability, THS) under baseline conditions (left) and mid‐century projections (right) for the terrestrial species analyzed. THS values range from 0 to 1 and represent temperature‐dependent feeding suitability normalized to each species' thermal optimum.

For the eight terrestrial insect species studied, both current and future scenarios highlight geographical variations projected feeding suitability under climate change conditions. In the current scenario, species such as *Hyles lineata* exhibit areas of high feeding suitability, primarily in North America, indicating strong activity or presence in these regions (Figure 4), reflecting temperature‐dependent feeding performance rather than inferred species occurrence or abundance. Conversely, *Cicindela hybrida* and *Formica schaufussi* display more varied consumption rates, with significant activity zones in Europe and Asia. Additionally, species such as *Messor pergandei* , *Ocymyrmex barbiger* , *Pogonomyrmex maricopa* , and *Pogonomyrmex rugosus* show distinct consumption patterns across different continents (Figure 4). Under future climate conditions, the maps suggest shifts or expansions in high‐consumption areas for several species, likely in response to changing environmental conditions. For instance, *Manduca sexta* shows an increase in red zones, indicating a possible expansion of temperature‐dependent feeding suitability under future conditions. Conversely, *Cicindela hybrida* experiences a reduction in blue zones, suggesting a potential decline in its feeding efficiency (Figure 4). Species‐specific percentage changes in the spatial extent of each Thermal Habitat Suitability (THS) class between baseline and future projections are summarized in Table S2.

**FIGURE 5:**
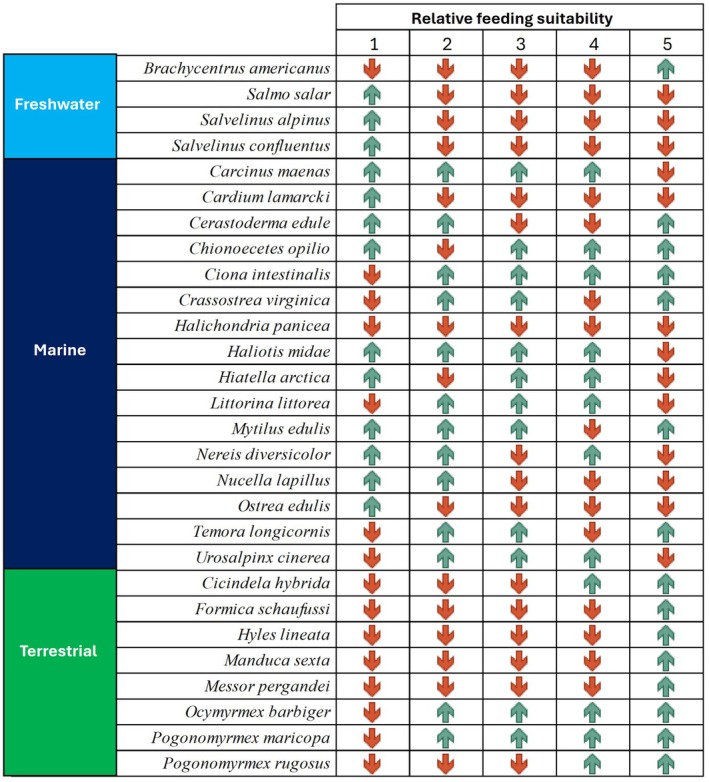
Changes in relative feeding suitability between current and future scenarios for the 28 species studied. The green arrows show an increase, the red a decrease in relative feeding suitability.

The changes in feeding efficiency between current and future scenarios reveal distinct trends (Figure 5). For freshwater species, all studied species exhibit an increase in feeding efficiency in category 1, while efficiency decreases in categories 2–5. For marine species, feeding efficiency trends vary across species. Some species, such as *Carcinus maenas* , show an increase in efficiency in categories 1 and 2, whereas others experience declines across multiple categories. For terrestrial species, feeding efficiency decreases across several categories for most species, although a few species show increases in specific categories, such as *Pogonomyrmex rugosus* in category 5.

**FIGURE 6:**
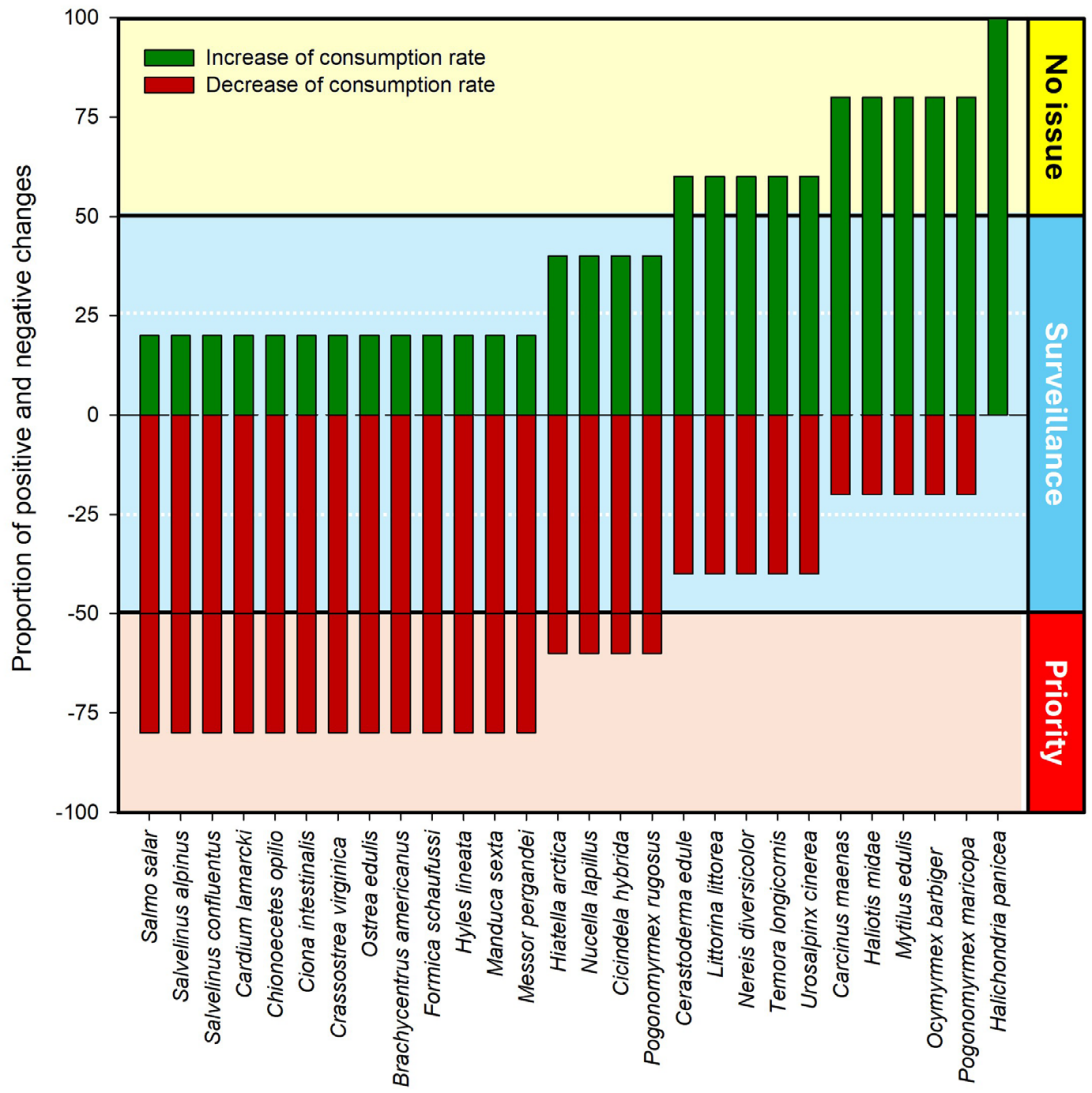
Screening‐based prioritization categories assigned to species based on the proportion of spatial change in relative feeding suitability (THS) between baseline and future scenarios. Range‐expanding status was defined as an increase in the spatial extent of areas with THS ≥ 0.6 between scenarios. Categories represent heuristic monitoring priorities rather than quantitative risk classes.

Based on the changes in consumption rates between current and future scenarios, variations across species were analyzed, highlighting both the positive and negative effects of climate change (Figure 6). Species were categorized into three priority zones based on the magnitude of these changes, with an additional distinction between range‐expanding and non‐expanding species to better capture their ecological impact. Here, “range‐expanding” species refer to taxa showing spatial increases in higher‐suitability THS classes between present and future projections, whereas “non‐expanding” species showed stable or contracting suitability distributions. Species like *Nereis diversicolor* and *Temora longicornis* show notable increases in consumption rates. While this may be beneficial for some non‐expanding species, range‐expanding species in this category may require closer monitoring to assess potential ecological disruptions (Table 2). Meanwhile, species like *Mytilus edulis* and *Halichondria panicea* maintain stable consumption rates, suggesting no immediate need for intervention. However, for range‐expanding species within this group, ongoing surveillance may still be necessary to track potential ecosystem shifts (Table 2).

**TABLE 2:** Ecological concern matrix for non‐expanding and range‐expanding species, which represents a heuristic interpretation framework linking observed changes in THS magnitude and range‐shift direction to relative monitoring priorities rather than a quantitative risk model.

Category	Non‐expanding species	Range‐expanding species
Priority	Strong decline or concern	Strong increase & potential invasiveness
Surveillance	Moderate changes requiring monitoring	Moderate increase, possible ecosystem shift
No issue	Stable consumption rates	Expansion with no major impact

*Note:* Range‐expanding status was determined based on increases in the spatial extent of areas with THS ≥ 0.6 between baseline and future projections.

## 4 Discussion

Our study presents a mechanistic application of thermal performance‐based modeling to assess the potential impacts of environmental temperature on species feeding efficiency at a global scale within a proof‐of‐concept framework. The integration of thermal performance curves into spatial projection workflows enables comparative screening of species responses based on their expected responses to climate change, providing a basis for identifying species or regions that may warrant further ecological monitoring and investigation. Our results indicate that feeding efficiency changes vary across species and ecosystems. Freshwater species in our dataset generally showed increases in projected feeding suitability, which is consistent with expectations from ectothermic metabolic scaling theory, where moderate warming toward thermal optima can enhance consumption rates until physiological limits are approached. In contrast, marine and terrestrial species exhibited more variable responses, likely reflecting broader differences in thermal niche position relative to projected warming trajectories. The tendency toward increased feeding suitability observed for several freshwater species likely reflects their current positioning below thermal optima across much of their modeled ranges. Because metabolic demand and consumption rates in ectotherms generally increase with temperature up to species‐specific optima (Pörtner 2010), moderate warming can strengthen interaction rates where present‐day temperatures remain suboptimal. Similar temperature‐driven increases in trophic interaction strength have been reported in temperate freshwater systems in both experimental and modeling studies (Dell et al. 2014), supporting the interpretation that projected efficiency gains reflect physiological scaling rather than simple spatial artifacts of the projection framework. These findings highlight the diverse ecological consequences of climate change on predator–prey dynamics and illustrate how temperature‐dependent feeding responses may contribute to understanding potential ecosystem‐level changes under warming scenarios, although additional validation and multi‐scenario analyses would be required before operational management application.

Temperature is widely recognized as the most significant variable influencing species movement, metabolism, and ecological responses in changing environments (Kordas et al. 2011). While other environmental factors, such as salinity, pH, and precipitation, may also have substantial impacts (Smyth and Elliott 2016), temperature remains the primary regulating factor in species life cycles, playing a crucial role in determining their seasonal occurrence and long‐term persistence (Schou and Cornwallis 2024). An organism's ability to respond to climate change is shaped by a combination of ecological, physiological, and genetic traits, along with its exposure to environmental stressors (Barrows et al. 2020). Since temperature is the primary abiotic factor influencing metabolism, particularly in ectothermic species (Pörtner 2010), climate warming—examined here under SSP5‐8.5 (CMIP6) scenario—acts as a dominant force that amplifies other environmental pressures (Jackson et al. 2021). In this study, temperature was used as the primary environmental driver because Thermal Performance Curves explicitly quantify temperature‐dependent physiological responses and provide a mechanistic basis for linking feeding efficiency to spatial projections. Other environmental variables such as precipitation, oxygen availability, or primary productivity were not included in the present framework because comparable trait‐based response functions were not consistently available across taxa and ecosystems at a global scale. Accordingly, the projections presented here represent a first‐order approximation of climate‐driven changes in feeding efficiency rather than a fully coupled multi‐driver ecological suitability model. Future work could extend this framework by integrating additional environmental constraints where trait‐response relationships are available. Anticipating future species range expansions is essential for assessing potential ecological disruptions and mitigating economic consequences.

In recipient communities, species proliferation due to rising temperatures could significantly alter ecosystem dynamics (Cardinale et al. 2006; Sarà et al. 2018). Such shifts may trigger cascading effects that compromise ecosystem functions and impact critical supporting and regulating services (Jax 2010; Baltar et al. 2019). This is particularly true for the model species examined in this study, as their distributions and predation effects are strongly temperature‐driven and influenced by both latitudinal and altitudinal gradients. For all genera considered, our predictions suggest the existence of temperature thresholds, as their projected range expansions and contractions, along with variations in consumption rates, differ significantly across temperature gradients (Figures 2, 3, 4) (Mohammadi et al. 2019; Ali et al. 2021). Regardless of the environment (freshwater, marine, or terrestrial), all modeled species have the potential to exert elevated predation pressures under projected range expansions, as suggested by temperature‐dependent feeding efficiency projections, which may influence top–down effects on native biota (Walne and Dean 1972; Gallepp 1977; Rissing 1982; Riisgard et al. 1992, Elliott and Elliott 2010). For example, among the modeled species, Atlantic salmon *Salmo salar* is known to impact both the environment and wild salmon populations by competing for food, space, and breeding partners. Additionally, its presence can lead to disease transmission and genetic introgression, further threatening native salmon stocks (Taranger et al. 2015). Similarly, in marine ecosystems, ragworm *Nereis diversicolor* influences benthic metabolism and nutrient dynamics, with its effects varying depending on sediment composition—altering processes in both organic‐poor and organic‐rich environments (Hansen and Kristensen 1998). In terrestrial ecosystems, harvester ants (*Messor* spp.) contribute to soil fertility enhancement by increasing microbial biomass and microbial activity in nest‐modified soils, ultimately affecting nutrient cycling and ecosystem functioning (Ginzburg et al. 2008).

Building on previous studies that integrated species‐specific thermal tolerance experiments with Bayesian‐based statistical approaches to model thermal performance (Mangano et al. 2020; Bosch‐Belmar et al. 2021), our study advances this framework by incorporating consumption data as a functional trait. This approach refines predictions of species responses by directly linking feeding performance to environmental conditions, allowing for a more ecologically informative assessment of species distribution and trophic interactions. While traditional functional species distribution models (F‐SDMs) have focused on mechanistic and correlative modeling to project ecological niches (Kearney and Porter 2009), our method improves ecological interpretability of temperature‐dependent feeding responses relative to temperature‐only suitability mapping by explicitly integrating species‐specific feeding dynamics into the modeling process. This approach conceptually addresses the mismatch between mechanistic functional trait data and georeferenced databases by integrating species‐specific feeding dynamics into spatial projections; however, no formal validation or cross‐validation was performed, and the extent to which mismatch is reduced remains to be quantitatively assessed (Bellwood et al. 2019). As food web structure is inherently linked to species distributions, this approach provides a more holistic perspective on ecological resilience and ecosystem shifts in response to climate change.

One of the key contributions of this study is the demonstration of a scalable methodology that can be adapted to specific assessment areas. While this research focused on a global‐scale application, the approach can be tailored to regional or ecosystem‐specific assessments, provided that adequate empirical data on prey availability and consumption rates are available. This flexibility makes it a valuable tool for environmental managers and policymakers tasked with prioritizing species for conservation or control efforts. Our model is particularly valuable for forecasting future changes in feeding efficiency under shifting temperature regimes. While we utilized temperature as the primary environmental proxy, the methodology is versatile and could incorporate other key environmental variables, such as salinity, precipitation, or pH, depending on the ecosystem and species of interest. This adaptability broadens its potential applications beyond thermal stress assessments.

Beyond feeding efficiency, this modeling approach holds potential for integrating alternative functional traits, such as functional response metrics or other ecological performance indicators. Additionally, it can be adapted to incorporate generic risk assessment outputs, making it a flexible tool for evaluating species' impacts under different environmental scenarios. By expanding the range of functional traits included in such models, future applications can enhance predictive capacity and improve management strategies tailored to specific conservation or resource management needs.

Despite the robustness of our approach, some key limitations must be acknowledged. One of the primary constraints is the lack of direct data on prey consumption patterns under predator influence for the examined species. In this study, we relied on experimental datasets that used the most common prey items, yet actual consumption rates may vary significantly in different environmental contexts. To refine future applications of this model, it is essential to incorporate localized prey availability data and experimentally derived consumption rates under varying thermal conditions. These observed patterns may, to some degree, be influenced by experimental constraints. In laboratory settings, thermal optima for key performance traits are typically determined under constant temperature conditions with organisms provided unlimited food resources. Notably, natural environments are far more dynamic, characterized by fluctuating temperatures and variable food availability. Since a lower food intake rate tends to reduce an organism's thermal optimum, estimates derived from controlled laboratory experiments may overestimate the true thermal optima observed in the wild (Elliott 1982). Consequently, laboratory‐derived thermal performance curves should be interpreted as estimates of physiological potential rather than realized field performance, and spatial projections based on these curves represent relative suitability patterns rather than direct predictions of interaction strength under natural conditions. Additionally, while TPCs provide valuable insights into species responses to temperature, they do not fully account for broader ecological interactions, such as competition, habitat availability, or human‐mediated changes. Another critical aspect to consider is the potential for spatial mismatches between predators and prey under climate change scenarios (Gomides et al. 2021). As species shift their distributions at different rates in response to warming, disruptions in predator–prey dynamics may occur, altering ecosystem stability (Carroll et al. 2024). In the present study, we do not analytically model prey distributions or predator–prey overlap, representing a conceptual limitation of the current framework. To address this, future research should incorporate predictive modeling of prey distributions alongside predator feeding efficiency assessments. This integration would enhance our ability to evaluate trophic interactions in a changing climate and improve conservation planning. Furthermore, the analyses were based on a relatively limited dataset, primarily due to the general lack of available data—particularly for freshwater fish species. As a result, while the patterns observed provide important insights, they should not be interpreted as globally representative. Consequently, observed trends should not be extrapolated to entire ecosystems or global scales, but rather illustrate the application of the proposed modeling framework. Broader data coverage, including underrepresented taxa and regions, is necessary to generalize the findings at a global scale. Finally, it is important to note that we did not formally propagate or combine uncertainties from Thermal Performance Curve fitting, climate model projections, emission scenarios, or spatial resolution. Consequently, the spatial projections and priority assessments presented here should be interpreted as first‐order, proof‐of‐concept illustrations rather than precise predictions. Future work could extend this framework to quantify and integrate these multiple sources of uncertainty, enabling more robust risk and management assessments.

### 4.1 Management Implications

From a management perspective, the predictive framework developed here provides a conceptual basis for exploring potential shifts in feeding efficiency across species under climate change. By assigning priority rankings based on projected changes in Thermal Habitat Suitability (THS), the framework allows comparative screening of species that may warrant further monitoring or investigation. We note that this classification does not incorporate species abundance, invasion status, or socio‐economic relevance, and the predictive skill of the framework has not been formally evaluated against real‐world case studies. Consequently, the outputs should not be interpreted as prescriptive management guidance. For example, while the tool can highlight species with higher projected feeding efficiency under warming scenarios, actual conservation or mitigation decisions would require additional context‐specific information, including prey availability, population dynamics, and ecological interactions. Despite these limitations, the framework demonstrates how temperature‐dependent feeding projections can inform hypothesis generation and comparative prioritization, providing a first‐order approach to identify species or regions where climate‐driven ecological changes may be most pronounced.

For example, the results obtained could be integrated into existing adaptive management frameworks, such as Natura 2000 management plans in Europe or risk assessments conducted by biosafety agencies. By identifying in advance the species likely to increase their trophic impact, our approach can feed into early warning systems and help direct resources toward priority actions (invasion control, conservation of vulnerable species).

## 5 Conclusion

Our findings highlight the conceptual potential of thermal performance‐based modeling as a proof‐of‐concept tool for exploring how climate change may affect species feeding efficiency. By identifying species likely to experience significant changes in feeding efficiency, this approach can support hypothesis generation and comparative screening, but the analysis is based on a limited dataset of 28 species and does not provide validated predictions at broader scales. While limitations exist, continued refinement of this methodology, including the incorporation of additional environmental variables and species interactions, will further enhance its applicability; claims of scalability or operational predictive use remain to be demonstrated through benchmarking and reproducibility tests. As such, this framework should be viewed as complementary to field monitoring and other decision‐support tools, primarily serving to prioritize species or regions for further study rather than as a stand‐alone management tool. Moving forward, interdisciplinary collaborations will be crucial in leveraging this tool to safeguard biodiversity and ecosystem stability in a changing world. Its potential lies primarily in its ability to prioritize and anticipate emerging pressures related to invasive species or declines in native predators.

## Author Contributions

**Guillaume Marchessaux:** conceptualization (supporting), data curation (equal), formal analysis (equal), investigation (supporting), methodology (lead), validation (equal), visualization (equal), writing – original draft (supporting), writing – review and editing (supporting). **Sadi Aksu:** data curation (equal), formal analysis (equal), methodology (supporting), validation (equal), visualization (equal), writing – review and editing (supporting). **Ali Serhan Tarkan:** conceptualization (lead), formal analysis (equal), investigation (lead), methodology (equal), supervision (lead), validation (supporting), writing – original draft (lead), writing – review and editing (supporting).

## Funding

The authors have nothing to report.

## Conflicts of Interest

The authors declare no conflicts of interest.

## Supporting information


**Data S1:** AICc table summary.

**Data S2:** R_codes.

**Figure S1:** Thermal performance curves obtained for the 3 Freshwater species studied.
**Figure S2:** Thermal performance curves obtained for the 16 Marine species studied.
**Figure S3:** Thermal performance curves obtained for the nine Terrestrial species studied.
**Table S1:** Models parameters, thermal limits of the species studied (CT_max_, critical threshold maximum; CT_min_, critical threshold minimum; T_opt_, optimal temperature).
**Table S2:** Percentage change in spatial extent of Thermal Habitat Suitability (THS) classes between baseline and future climate projections for each species across freshwater, marine, and terrestrial habitats.

## Data Availability

All the required data are uploaded as [Link], [Link], [Link].
