# Supplementary material for: A Functional Trait‐Based Approach to Mapping Climate‐Driven Changes in Temperature‐Dependent Feeding Suitability
Source: Ecol Evol. 2026 May 3;16(5):e73623. doi: 10.1002/ece3.73623 (PMC13135894; doi:10.1002/ece3.73623)
Supplement: Supplementary file 3 — Figure S1: Thermal performance curves obtained for the 3 Freshwater species studied. Figure S2: Thermal performance curves obtained for the 16 Marine species studied. Figure S3: Thermal performance curves obtained for the nine Terrestrial species studied. Table S1: Models parameters, thermal limits of the species studied (CTmax, critical threshold maximum; CTmin, critical threshold minimum; Topt, optimal temperature). Table S2: Percentage change in spatial extent of Thermal Habitat Suitability (THS) classes between baseline and future climate projections for each species across freshwater, marine, and terrestrial habitats. [file ECE3-16-e73623-s001.docx]

**Supplementary tables**

**Table S1.** Models parameters, thermal limits of the species studied (T_opt_: optimal temperature ; CT_min_: critical threshold minimum, CT_max_: critical threshold maximum).

| Habitat | Species | Best model | Topt | CTmin | CTmax |
| --- | --- | --- | --- | --- | --- |
| Freshwater | *Salmo salar* | Ratkowsky | 18.98 | 4.23 | 26.69 |
| Freshwater | *Salvelinus alpinus* | Gaussian | 14.6 | 1.47 | 27.74 |
| Freshwater | *Salvelinus confluentus* | Gaussian | 13.28 | 2.16 | 24.4 |
| Marine | *Carcinus maenas* | Rezende | 16.68 | 2.5 | 18.67 |
| Marine | *Cardium lamarcki* | Modifiedgaussian | 21.99 | 12.5 | 31.48 |
| Marine | *Cerastoderma edule* | Gaussian | 15.54 | -8.42 | 39.51 |
| Marine | *Chionoecetes opilio* | Boatman | 5.29 | -4.9 | 14.12 |
| Marine | *Ciona intestinalis* | Ratkowsky | 20.76 | -1.31 | 22.84 |
| Marine | *Crassostrea virginica* | Gaussian | 28.31 | -0.58 | 57.21 |
| Marine | *Halichondria panicea* | Lactin2 | 14.7 | 3.34 | 15.04 |
| Marine | *Haliotis midae* | Rezende | 20.89 | -5.97 | 24.84 |
| Marine | *Hiatella arctica* | Pawar | 15.97 | -2.25 | 21.17 |
| Marine | *Littorina littorea* | Flinn | 24.9 | -38.12 | Inf |
| Marine | *Mytilus edulis* | Quadratic | 15.46 | -0.35 | 31.27 |
| Marine | *Nereis diversicolor* | Ratkowsky | 18.39 | -2.6 | 26.03 |
| Marine | *Nucella lapillus* | Sharpeschoolfull | 21.57 | 2.76 | 24.56 |
| Marine | *Ostrea edulis* | Modifiedgaussian | 21.06 | 14.39 | 27.74 |
| Marine | *Temora longicornis* | Quadratic | 15.46 | -0.35 | 31.27 |
| Marine | *Urosalpinx cinerea* | Gaussian | 25.51 | 8.26 | 42.76 |
| Terrestrial | *Brachycentrus americanus* | Johnson_lewin | 22.84 | Inf | 32.37 |
| Terrestrial | *Cicindela hybrida* | Gaussian | 39.06 | 13.74 | 64.37 |
| Terrestrial | *Formica schaufussi* | Beta | 27.85 | 15.59 | 39.07 |
| Terrestrial | *Hyles lineata* | Quadratic | 24.25 | 7.42 | 41.08 |
| Terrestrial | *Manduca sexta* | Quadratic | 23.58 | 5.93 | 41.23 |
| Terrestrial | *Messor pergandei* | Lactin2 | 35.21 | 7.13 | 39.59 |
| Terrestrial | *Ocymyrmex barbiger* | Gaussian | 57.69 | 19.32 | 96.05 |
| Terrestrial | *Pogonomyrmex maricopa* | Gaussian | 40.56 | 14.18 | 66.94 |
| Terrestrial | *Pogonomyrmex rugosus* | Rezende | 44.27 | 6.14 | 51.65 |

**Table S2.** Percentage change in spatial extent of Thermal Habitat Suitability (THS) classes between baseline and future climate projections for each species across freshwater, marine, and terrestrial habitats.

| **Habitat** | **Species** | **Change Class 1** | **Change Class 2** | **Change Class 3** | **Change Class 4** | **Change Class 5** |
| --- | --- | --- | --- | --- | --- | --- |
| Freshwater | *Salmo salar* | 27.2 | -70.5 | -73.4 | -68.1 | -74.5 |
| Freshwater | *Salvelinus alpinus* | 13.2 | -29.9 | -26.0 | -18.9 | -21.6 |
| Freshwater | *Salvelinus confluentus* | 9.6 | -21.4 | -19.0 | -21.3 | -17.8 |
| Freshwater | *Brachycentrus americanus* | -82.8 | -56.4 | -43.4 | -9.2 | 28.5 |
| Marine | *Carcinus maenas* | 6439.3 | 2350.3 | 343.8 | 11.0 | -1.3 |
| Marine | *Cardium lamarcki* | 15.3 | -4.1 | -18.3 | -19.0 | -13.7 |
| Marine | *Cerastoderma edule* | 12.3 | 1.2 | -10.1 | -2.7 | 0.4 |
| Marine | *Chionoecetes opilio* | 3.5 | -16.1 | 5.9 | 6.1 | 3.3 |
| Marine | *Ciona intestinalis* | -0.1 | 7120.7 | 8274.1 | 2093.0 | 2066.1 |
| Marine | *Crassostrea virginica* | -6.5 | 7.2 | 1.7 | -9.5 | 6.8 |
| Marine | *Halichondria panicea* | -11.4 | -10.8 | -10.7 | -10.8 | -4.4 |
| Marine | *Haliotis midae* | 439.8 | 41.2 | 11.0 | 13.7 | -3.0 |
| Marine | *Hiatella arctica* | 0.2 | -2.8 | 6.2 | 1.1 | -0.3 |
| Marine | *Littorina littorea* | -7.2 | 3.7 | 3.3 | 102.4 | -12.1 |
| Marine | *Mytilus edulis* | 910.6 | 425.1 | 301.0 | -22.8 | 40.2 |
| Marine | *Nereis diversicolor* | 3.2 | 1.1 | -0.6 | 0.3 | -6.5 |
| Marine | *Nucella lapillus* | 1.8 | 3.1 | -1.2 | -8.0 | -11.6 |
| Marine | *Ostrea edulis* | 8.1 | -20.7 | -13.1 | -11.6 | -11.3 |
| Marine | *Temora longicornis* | -28.5 | 22.2 | 0.8 | -4.5 | 3.5 |
| Marine | *Urosalpinx cinerea* | -4.2 | 1.9 | 10.1 | 84.8 | -17.6 |
| Terrestrial | *Cicindela hybrida* | -18.2 | -30.9 | -43.3 | 130.9 | 1104.7 |
| Terrestrial | *Formica schaufussi* | -9.6 | -41.8 | -47.5 | -43.1 | 24.2 |
| Terrestrial | *Hyles-lineata* | -12.4 | -15.8 | -9.8 | -1.4 | 6.6 |
| Terrestrial | *Manduca sexta* | -11.9 | -16.7 | -9.3 | -2.0 | 6.7 |
| Terrestrial | *Messor pergandei* | -100.0 | -100.0 | -99.9 | -99.2 | 244.8 |
| Terrestrial | *Ocymyrmex barbiger* | -8.1 | 18.7 | 30.6 | 70.2 | 154.4 |
| Terrestrial | *Pogonomyrmex rugosus* | -17.7 | -32.9 | -31.7 | 236.4 | 1040.0 |
| Terrestrial | *Pogonomyrmex maricopa* | -12.6 | 2.8 | 68.3 | 61.5 | 643.4 |

**Supplementary figures**


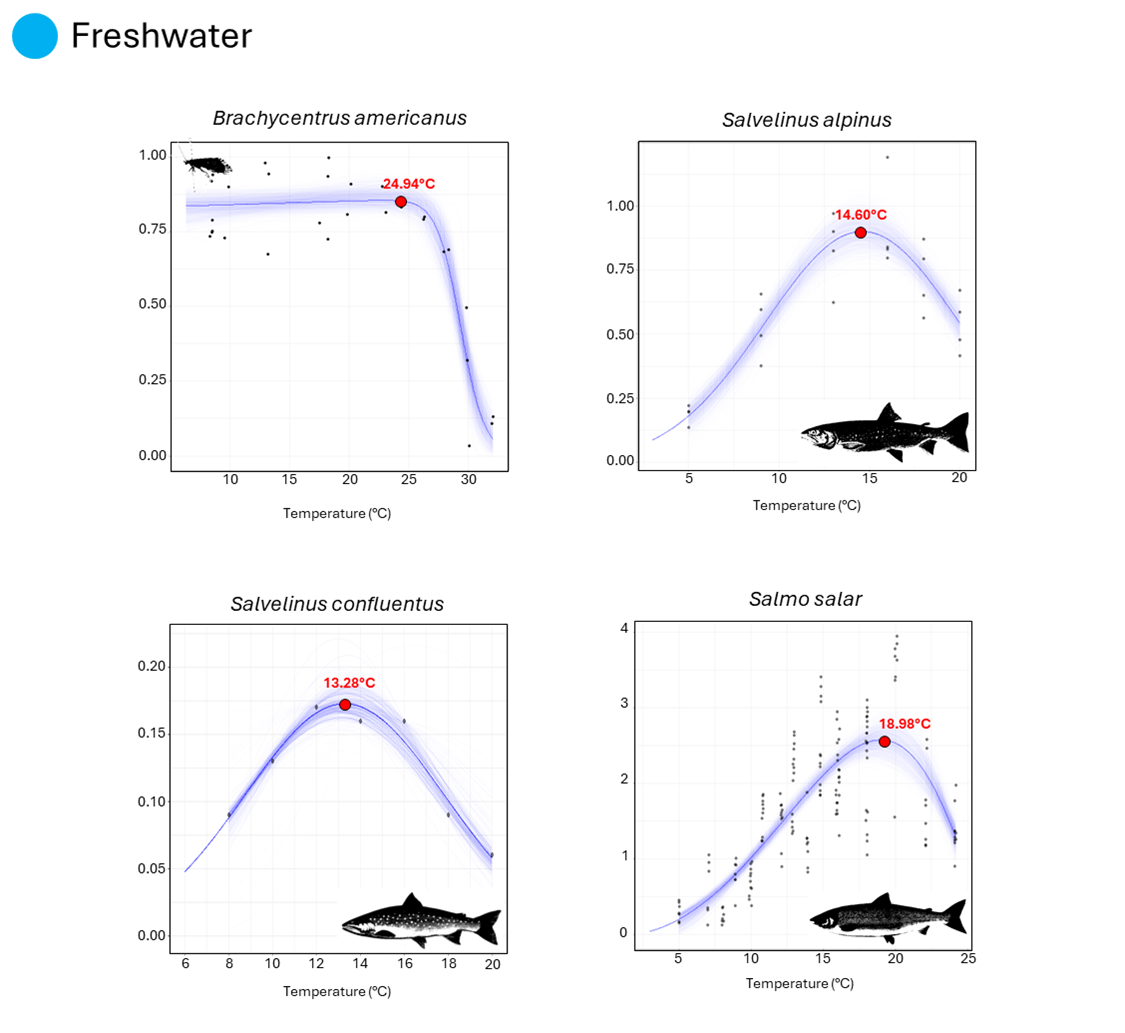


**Supplementary Figure 1**. Thermal performance curves obtained for the 3 Freshwater species studied.


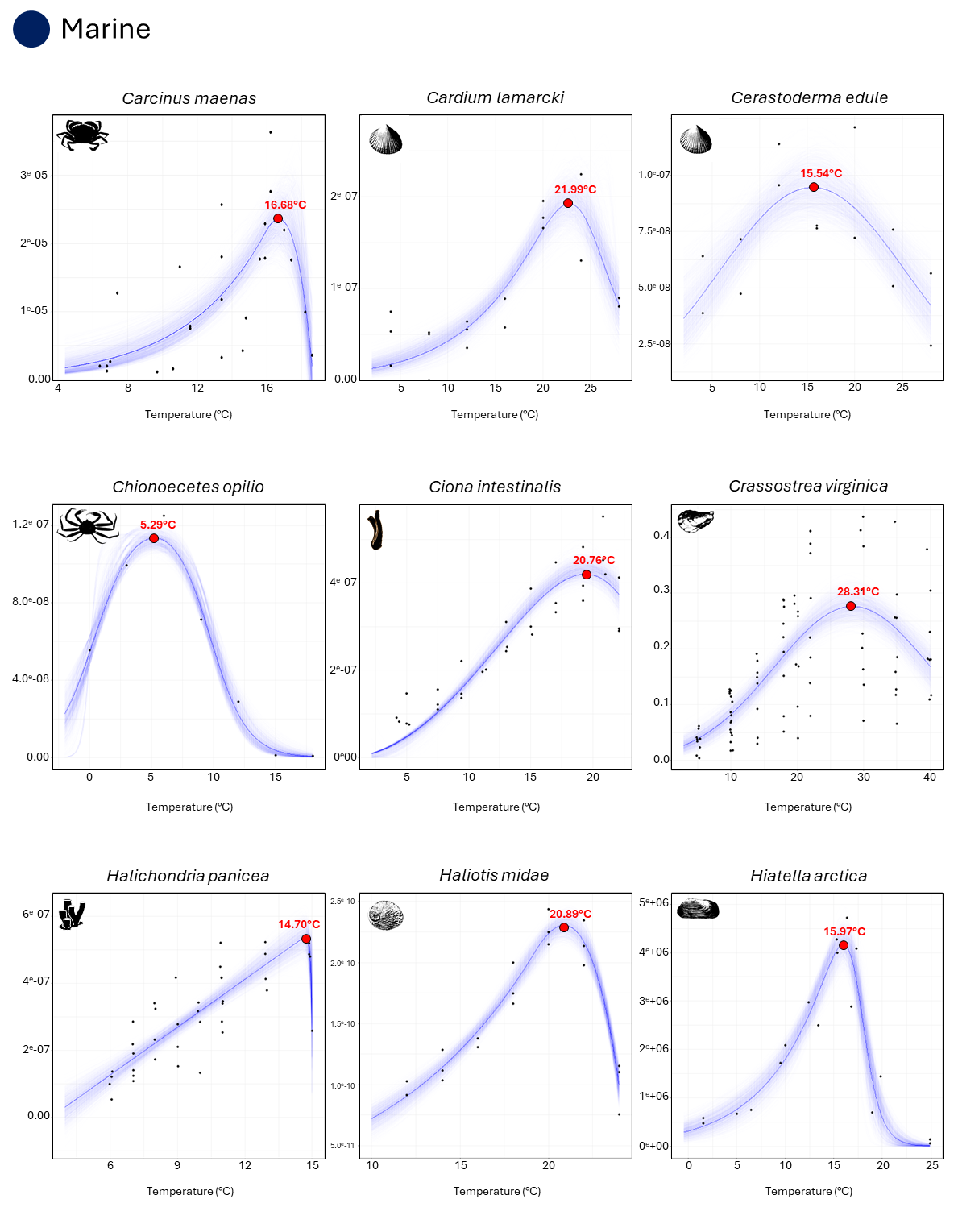


**Supplementary Figure 2**. Thermal performance curves obtained for the 16 Marine species studied.


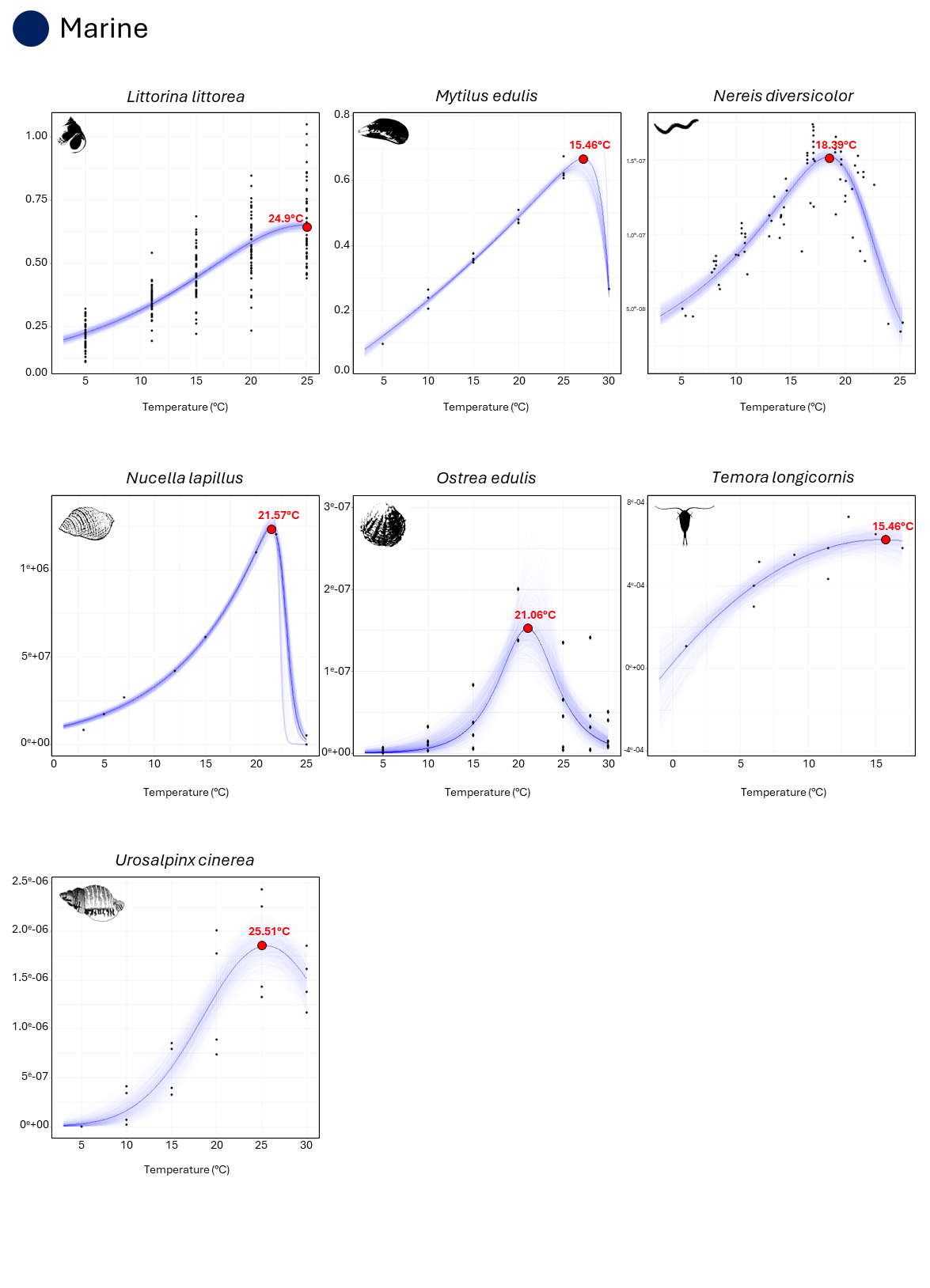


**Supplementary Figure 2 (continue)**


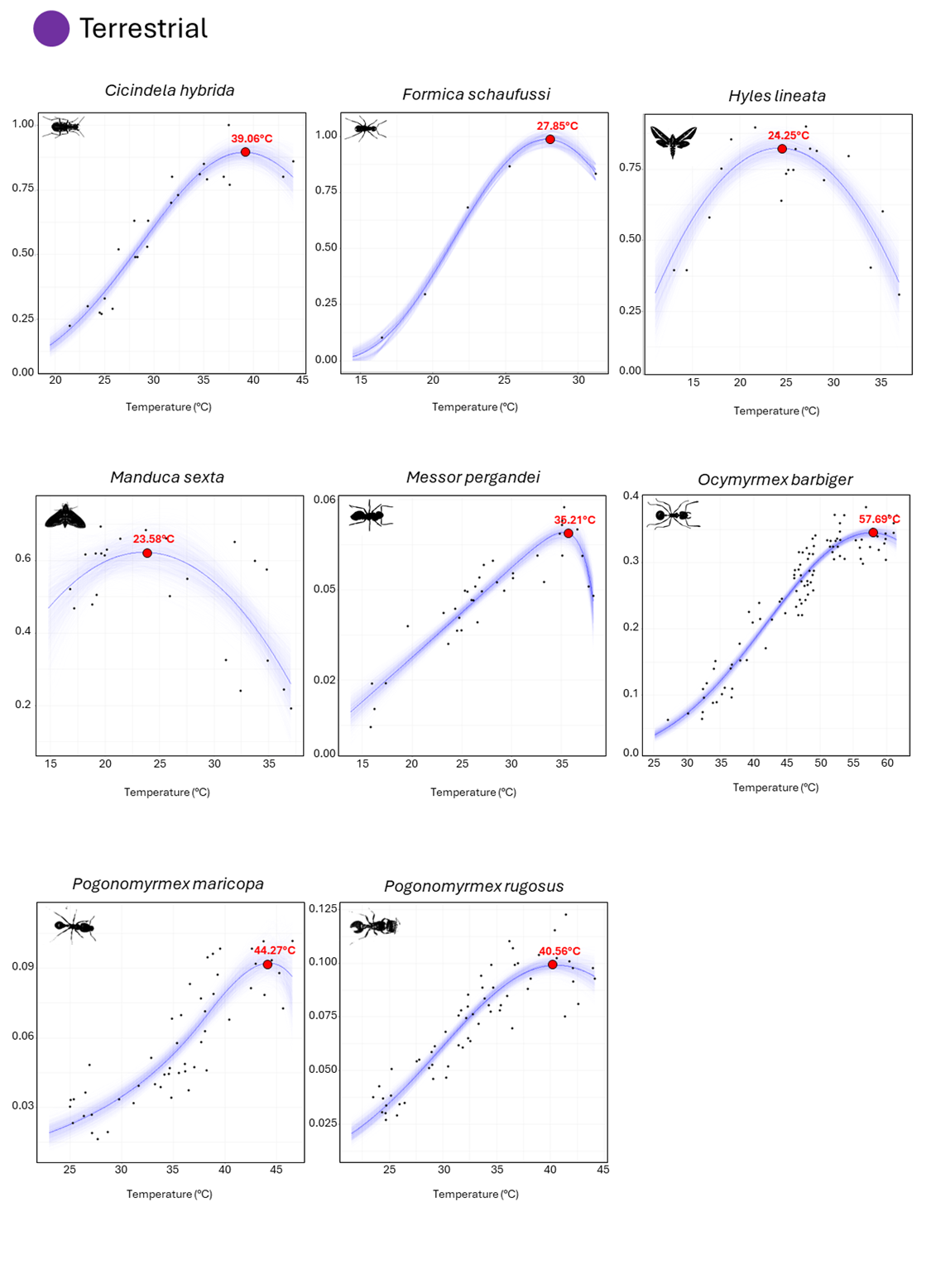


**Supplementary Figure 3**. Thermal performance curves obtained for the 9 Terrestrial species studied.
